# Autoantibody and Cytokine Profiles during Treatment with Belimumab in Patients with Systemic Lupus Erythematosus

**DOI:** 10.3390/ijms21103463

**Published:** 2020-05-14

**Authors:** Ioannis Parodis, Emil Åkerström, Christopher Sjöwall, Azita Sohrabian, Andreas Jönsen, Alvaro Gomez, Martina Frodlund, Agneta Zickert, Anders A Bengtsson, Johan Rönnelid, Iva Gunnarsson

**Affiliations:** 1 Division of Rheumatology, Department of Medicine, Karolinska Institutet, SE-171 76 Stockholm, Sweden; emil.aakerstroem@gmail.com (E.A.); alvarogomezg@ug.uchile.cl (A.G.); agneta.zickert@sll.se (A.Z.); iva.gunnarsson@sll.se (I.G.); 2 Rheumatology, Karolinska University Hospital, SE-171 76 Stockholm, Sweden; 3 Rheumatology/Division of Inflammation and Infection, Department of Biomedical and Clinical Sciences, Linköping University, SE-581 85 Linköping, Sweden; christopher.sjowall@liu.se (C.S.); martina.frodlund@regionostergotland.se (M.F.); 4 Department of Immunology, Genetics and Pathology, Uppsala University, SE-751 85 Uppsala, Sweden; azita.sohrabian@igp.uu.se (A.S.); johan.ronnelid@igp.uu.se (J.R.); 5 Rheumatology, Department of Clinical Sciences Lund, Lund University, Skåne University Hospital, SE-222 42 Lund, Sweden; andreas.jonsen@med.lu.se (A.J.); anders.bengtsson@med.lu.se (A.A.B.)

**Keywords:** systemic lupus erythematosus, cytokines, autoantibodies, immune complexes, autoimmunity, biologic therapies, B cells

## Abstract

We investigated whether belimumab treatment impacts on levels of autoantibodies and cytokines of interest in systemic lupus erythematosus (SLE). Longitudinally collected serum samples from 78 belimumab-treated Swedish SLE patients were analysed. Serum cytokine levels were determined using Luminex xMAP technology, and nuclear antigen autoantibody specificities using addressable laser bead immunoassay. In patients with detectable levels at baseline, interferon (IFN)-α2 levels were lower at month 6 (median; interquartile range (IQR): 8.9; 1.5–54.9 pg/mL) versus baseline (28.4; 20.9–100.3 pg/mL; *p* = 0.043). Interleukin (IL)-6 (baseline: 7.1; 2.9–16.1 pg/mL) decreased from month 6 (0.5; 0.5–6.3 pg/mL; *p* = 0.018) and throughout a 24 month follow-up. IL-10 (baseline: 12.6; 2.8–29.7 pg/mL) showed more rapid decreases from month 3 (1.8; 0.6–9.1 pg/mL; *p* = 0.003). Levels of anti-dsDNA (*p* < 0.001), anti-Smith antigen (Sm) (*p* = 0.002), anti-U1 small nuclear ribonucleoprotein (U1RNP) (*p* < 0.001), anti-Sm-U1RNP complex (*p* = 0.028), and anti-ribosomal P (*p* = 0.012) antibodies decreased from month 3 and remained decreased. Anti-Sm positivity at baseline was associated with higher probability and/or shorter time to achieve sustained SLE responder index-4 response (hazard ratio (HR): 2.52; 95% CI: 1.20–5.29; *p* = 0.015), independently of other factors. Decline of IL-6 levels through month 3 was greater in responders. In summary, belimumab treatment lowered IFN-α2, IL-6, and IL-10 levels, as well as levels of multiple autoantibodies, however after different time spans. Notably, anti-Sm positivity and early decline in IL-6 levels were associated with favorable treatment outcome.

## 1. Introduction

Belimumab, a monoclonal antibody against the soluble counterpart of B cell activating factor (BAFF), also known as B lymphocyte stimulator (BLyS), is used for the treatment of active systemic lupus erythematosus (SLE) despite standard of care therapy, that is, antimalarial agents and non-selective immunosuppressants including glucocorticoids [1]. The effects of belimumab on serum levels of anti-double stranded (ds)DNA antibodies and BAFF, as well as the homologous to BAFF plasma cell survival factor a proliferation-inducing ligand (APRIL), have been demonstrated in previous studies [2,3,4]. Furthermore, predictors of response and non-response to belimumab treatment have been implicated; high disease activity and anti-dsDNA positivity at baseline have been associated with increased probability of good response, whereas established organ damage, especially in the cardiovascular and neuropsychiatric domains, has been shown to reduce belimumab efficacy [4,5,6,7,8].

In a recent study, we compared free circulating serum levels of multiple autoantibody specificities with their corresponding levels in circulating immune complexes (IC), and demonstrated that high anti-dsDNA antibody levels in IC, but not in serum, prior to belimumab treatment initiation were associated with clinical improvement [9]. Importantly, B cells also have functions other than antibody production [10], such as antigen presentation and proinflammatory cytokine excretion [11,12], and cell types other than B cells also express receptors for BAFF [13], including certain subsets of T cells [14]. Thus, belimumab may be expected to also exert indirect effects on the equilibrium of immune responses in SLE, warranting thorough survey of its impact on SLE-associated immune pathways.

In the present study, we investigated whether belimumab treatment impacts levels of cytokines outside the BAFF/APRIL pathway but implicated in SLE pathogenesis and lupus drug development, as well as nuclear antigen autoantibody specificities commonly used for diagnosis and surveillance. We also investigated the performance of detectable cytokine levels and autoantibody positivity at baseline as predictors of response to belimumab treatment.

## 2. Results

**Figure 1:**
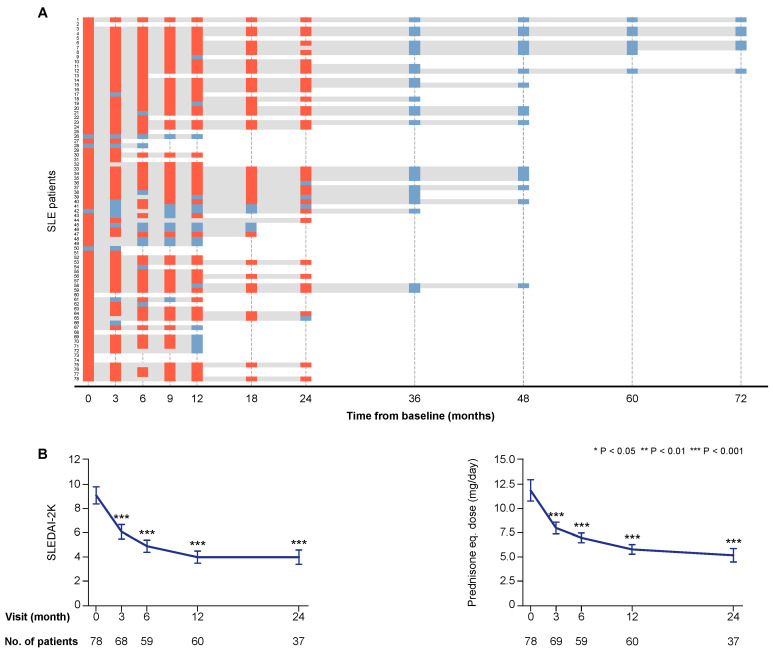
Follow-up and disease activity over time. Panel (**A**) illustrates the individual follow-up time for each one of the patients. The bars in grey represent time on treatment. The orange rectangles correspond to follow-up visits when a clinical assessment was performed and serum samples were available for analysis. The light blue rectangles correspond to follow-up visits from which clinical data only were obtained. The charts in panel (**B**) illustrate systemic lupus erythematosus (SLE) disease activity according to SLE Disease Activity Index 2000 (SLEDAI-2K) scores, and prednisone equivalent dose over time on treatment with belimumab. The blue lines connect mean values of the distributions at given time points, and the whiskers represent standard errors of the mean. The number of patients contributing to the respective measurement is indicated. Asterisks indicate statistically significant changes compared with baseline, on the basis of the non-parametric paired Wilcoxon signed rank test.

Seventy-three patients (93.6%) were women in line with the general estimation of the female-to-male ratio of SLE patients in Sweden [15], and the median age at the time of enrolment was 41.3 years (interquartile range (IQR): 31.6–51.4 years). The clinical course of the first 55 patients enrolled in the study has been reported previously [4]. The total follow-up time for each study participant, as well as SLE Disease Activity Index 2000 (SLEDAI-2K) scores and prednisone equivalent doses during treatment with belimumab are presented in Figure 1.

**Figure 2:**
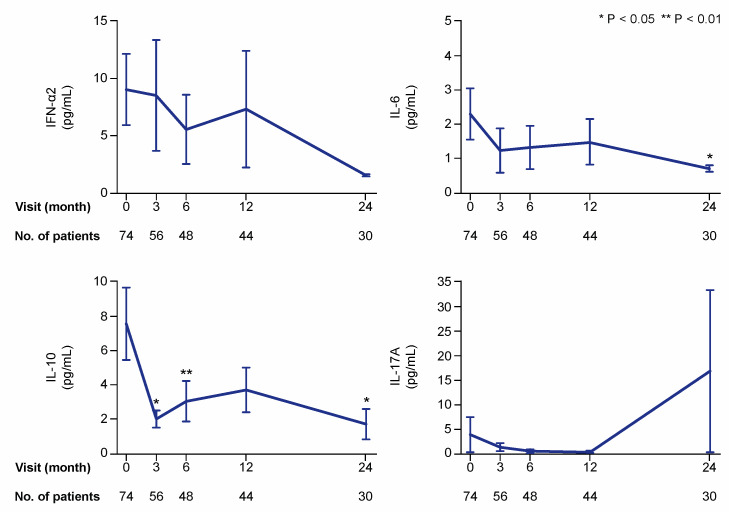
Selected cytokines during treatment with belimumab. The charts illustrate serum levels of selected cytokines—interferon (IFN)-α2, interleukin (IL)-6, IL-10, and IL-17A—at specific time points during treatment with belimumab. The blue lines connect mean values of the distributions at the different times points, and the whiskers represent standard errors of the mean. The numbers of patients contributing to the measurements are indicated. Asterisks indicate statistically significant changes compared with baseline, on the basis of the non-parametric paired Wilcoxon signed rank test.

**Figure 3:**
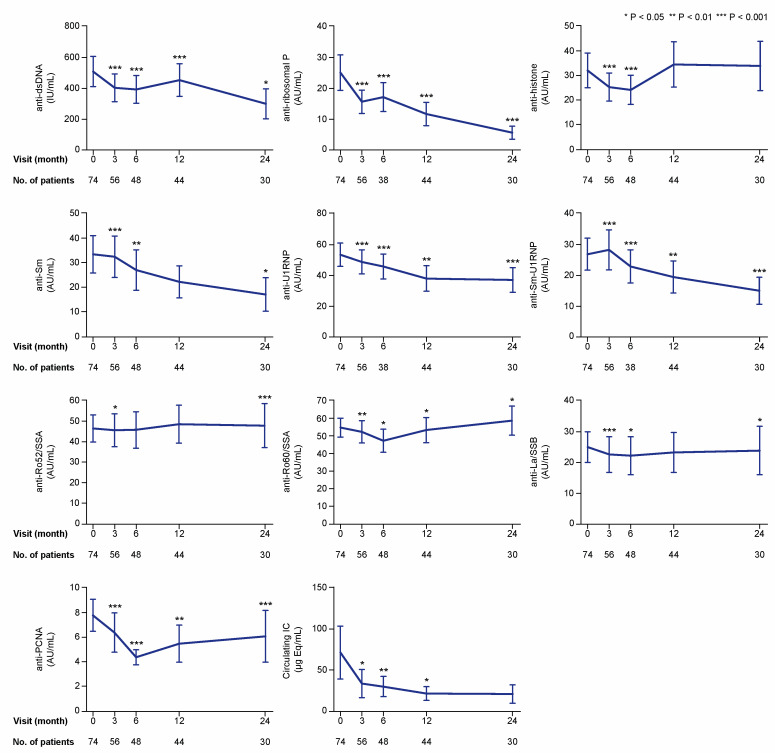
Autoantibody specificities and IC during treatment with belimumab. The charts illustrate serum levels of multiple nuclear antigen autoantibody specificities at specific time points during treatment with belimumab. The blue lines connect mean values of the distributions at the different times points, and the whiskers represent standard errors of the mean. The numbers of patients contributing to the measurements are indicated. Asterisks indicate statistically significant changes compared with baseline, based on the non-parametric paired Wilcoxon signed rank test. IC: immune complex; IU: international units; AU: arbitrary units.

**Figure 4:**
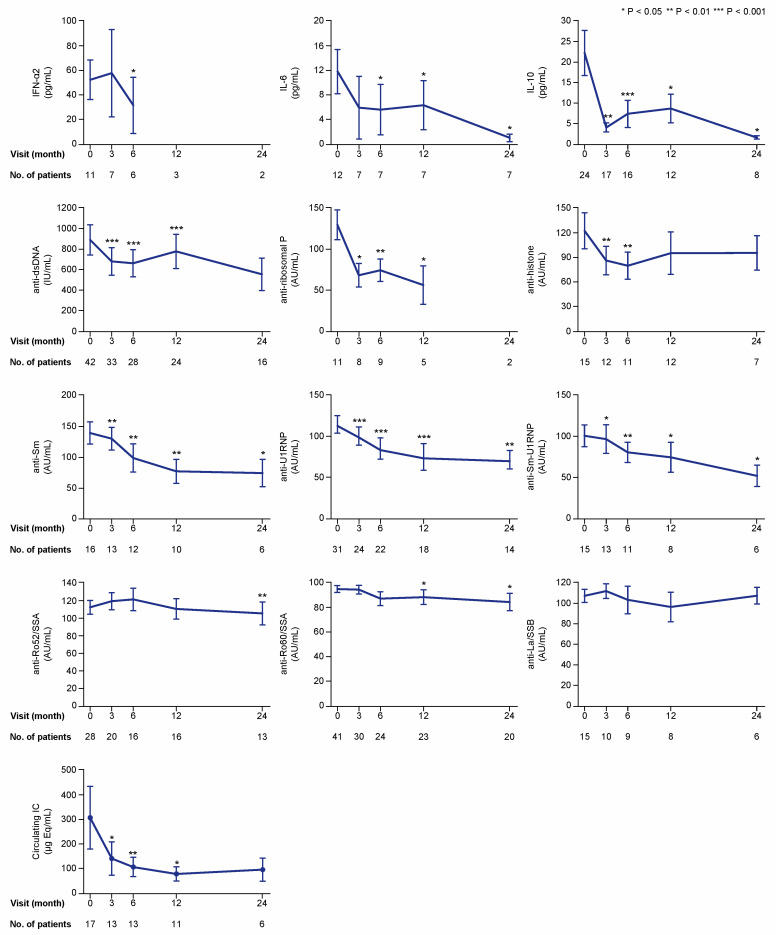
Cytokine, autoantibody, and immune complex (IC) levels if detectable or positive at baseline. The charts illustrate serum levels of selected cytokines and nuclear antigen autoantibody specificities over time on belimumab treatment in patients with detectable levels for cytokines and levels above the threshold for positivity for autoantibodies and immune complexes. The blue lines connect mean values of the distributions at the different times points, and the whiskers represent standard errors of the mean. The numbers of patients contributing to the measurements are indicated; data samples with fewer than five patients were dispensed from statistical analysis and graphical illustration. Asterisks indicate statistically significant changes compared with baseline, on the basis of the non-parametric paired Wilcoxon signed rank test. IC: immune complex; IFN: interferon; IL: interleukin; IU: international units; AU: arbitrary units.

We first analysed and graphically illustrated data for all patients regarding serum levels of the examined cytokines (Figure 2), autoantibodies, and circulating IC (Figure 3) over time on belimumab therapy. We next analysed serum levels of the examined cytokines, autoantibodies, and circulating IC during belimumab treatment in patients with detectable (for cytokines) or positive (for autoantibodies and IC) levels at baseline (Figure 4). Serum levels of autoantibody specificities in a proportion of patients and follow-up time from the same cohort have been included in previous investigations [4,9,16]. With regard to patients with undetectable cytokine levels, or negative autoantibody or circulating IC levels at baseline, we only observed a few seroconversions to detectable or positive levels, respectively, during the 24 month follow-up. These are reported in detail in Table S1.

### 2.1. Cytokine Levels during Belimumab Therapy

In the first analysis including all patients (also patients with cytokine levels below the lower detection limit of the assay), serum levels of interleukin (IL)-10 showed the most prominent changes over time, with statistically significant decreases as soon as at the 3 month follow-up (mean: 2.0; median 0.6; IQR: 0.6–0.6 pg/mL) compared with baseline (mean: 7.6; median 0.6; IQR: 0.6–2.8 pg/mL; *p* = 0.016). Serum levels of IL-6 (baseline mean: 2.3; median 0.5; IQR: 0.5–0.5 pg/mL) showed a slower decline, which reached statistical significance at month 24 (mean: 0.7; median 0.5; IQR: 0.5–0.5 pg/mL; *p* = 0.043). Changes in levels of interferon (IFN)-α2 and IL-17A did not reach statistical significance in this analysis (Figure 2).

At baseline, the number of patients with detectable levels of IFN-α2, IL-10, and IL-6 was 11, 24, and 12, respectively (Figure 4). Because only one patient had detectable levels of IL-17A, this cytokine was excluded from further analysis. In the analysis of patients with detectable baseline levels, serum levels of IFN-α2 were lower at month 6 (median: 8.9; IQR: 1.5–54.9 pg/mL) compared with baseline (median: 28.4; IQR: 20.9–100.3 pg/mL; *p* = 0.043), but not at month 3 (*p* = 0.345). Levels of IL-6 showed decreases from baseline (median: 7.1; IQR: 2.9–16.1 pg/mL) to month 6 (median: 0.5; IQR: 0.5–6.3 pg/mL; *p* = 0.018) and throughout a 24 month follow-up. Levels of IL-10 (baseline median: 12.6; IQR: 2.8–29.7 pg/mL) showed more rapid decreases at month 3 (median: 1.8; IQR: 0.6–9.1 pg/mL; *p* = 0.003) and remained significantly lower than baseline levels over a 24 month follow-up (Figure 4).

### 2.2. Autoantibody and IC Levels during Belimumab Therapy

In the first analysis including all patients, serum levels of anti-dsDNA showed profound decreases from baseline values (median: 82.8; IQR: 11.7–499.5 international units (IU)/mL), reaching statistical significance at month 3 (median: 63.9; IQR: 10.1–588.3 IU/mL; *p* < 0.001), which was maintained throughout a 24 month follow-up (Figure 3). Serum levels of anti-Smith antigen (Sm) levels also decreased over time compared with baseline levels (median: 2.7; IQR: 0.6–19.7 arbitrary units (AU)/mL); these decreases were statistically significant at the 3 month visit (median: 1.8; IQR: 0.5–18.1 AU/mL; *p* < 0.001) and remained significantly decreased throughout a 24 month follow-up, with the exception of the 12 month visit (*p* = 0.145). Levels of anti-U1 small nuclear ribonucleoprotein (U1RNP) were significantly decreased compared with baseline levels (median: 17.8; IQR: 3.0–86.1 AU/mL) at month 3 and throughout the follow-up period until the 24 month visit (median: 14.7; IQR: 1.4–59.4 AU/mL; *p* < 0.001). Similarly, levels of antibodies against the Sm-U1RNP complex were decreased compared with baseline at all studied follow-up time points (Figure 3). Serum levels of circulating IC showed decreases compared with baseline levels (median: 1.2; IQR: 0.1–10.1 μg Eq/mL) at month 3 (median: 0.7; IQR: 0.1–9.8 μg Eq/mL; *p* = 0.031), and remained decreased at month 6 (*p* = 0.009) and 12 (*p* = 0.049), but not at month 24 (*p* = 0.272).

Numbers of patients with serum autoantibody levels above the thresholds for positivity at baseline were sufficient for further analysis for most of the antibody specificities, that is, anti-dsDNA (*n* = 42), anti-histone (*n* = 15), anti-Sm (*n* = 16), anti-Sm-U1RNP (*n* = 15), anti-U1RNP (*n* = 31), anti-ribosomal P (*n* = 11), anti-Ro52/SSA (*n* = 28), anti-Ro60/SSA (*n* = 41), and anti-La/SSB (*n* = 15). However, only two patients had positive levels of antibodies against proliferating cell nuclear antigen (anti-PCNA), and this specificity was therefore not included in the subsequent analyses. In patients with positive baseline levels, levels of anti-dsDNA (*p* < 0.001), anti-Sm (*p* = 0.002), anti-Sm-U1RNP (*p* = 0.028), anti-U1RNP (*p* < 0.001), and anti-ribosomal P (*p* = 0.012) antibodies were found to be reduced at month 3 and remained significantly lower than baseline levels over the 24 month study period (Figure 4). Anti-histone antibody levels showed decreases at month 3 (*p* = 0.008) and 6 (*p* = 0.003) from treatment initiation, but were not significantly changed compared with baseline levels at later time points. In patients with baseline circulating IC levels equal to or above the threshold for positivity (10.8 μg Eq/mL) (*n* = 17), serum IC levels showed decreases compared with baseline levels (median: 76.5; IQR: 30.1–278.3 μg Eq/mL) at month 3 (median: 28.1; IQR: 18.6–180.3 μg Eq/mL; *p* = 0.028), and remained decreased at month 6 (*p* = 0.009) and 12 (*p* = 0.021), but no significant change was observed at month 24 (*p* = 0.345; Figure 4).

### 2.3. Autoantibody to total IgG ratios

To investigate whether the decreases in autoantibody levels were specific for the respective autoantibody or reflected a general decrease in IgG levels, we assessed autoantibody to total IgG ratios over time on treatment. Because data on total IgG were available in a limited proportion of the study population (*n* = 55), and only for the early follow-up period in the majority of these patients, we only investigated changes from baseline to month 6. The median of total IgG levels decreased by 5.1% (*p* = 0.001). Among autoantibody specificities, anti-dsDNA to total IgG ratios declined from a median of 4.5 (IQR: 0.7–24.5) IU/mg to a median of 3.6 (IQR: 0.7–31.1) IU/mg (*p* = 0.002), anti-ribosomal P to total IgG ratios declined from a median of 0.14 (IQR: 0.08–0.60) AU/mg to a median of 0.10 (IQR: 0.08–0.38) AU/mg (*p* < 0.001), and anti-PCNA to total IgG ratios declined from a median of 0.20 (IQR: 0.13–0.33) AU/mg to a median of 0.16 (IQR: 0.10–0.26) AU/mg (*p* = 0.004), whereas the rest of the autoantibody to total IgG ratios did not differ significantly between baseline and month 6 (*p* = ns for all). 

### 2.4. Baseline Cytokine, Autoantibody, and IC Profiles as Predictors of Treatment Response

We evaluated the patients’ baseline cytokine (levels above the detection limit of the assay), autoantibody (levels above the threshold for positivity), and IC (positive levels) status with regard to achievement of different outcomes; IL-17A and anti-PCNA were excluded from this analysis due to low numbers of eligible patients.

**Figure 5:**
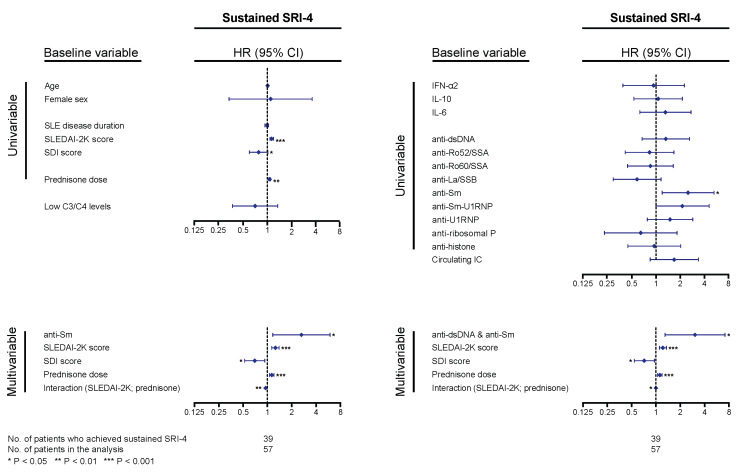
Associations with systemic lupus erythematosus responder index 4 (SRI-4) response. The forest plots illustrate results from proportional hazards regression (Cox regression) models created to explore potential associations between baseline levels of cytokines, autoantibodies, or circulating immune complexes (IC) and achievement of sustained SRI-4 response (fulfilment of the SRI-4 conditions at two consecutive follow-up visits, at least 3 months apart). Demographic and disease-specific factors were also analyzed for the purpose of adjustments for confounding potentiality. Time in the models represents the time from baseline to the first follow-up visit when sustained SRI-4 was achieved for patients who achieved the outcome, and total follow-up time for patients who did not. All baseline variables were first tested in univariable (simple) models, and variables showing significant associations with attainment (or non-attainment) of SRI-4 were next included in a multivariable model. Next, we substituted anti-Smith antigen (Sm) positivity with double positivity for anti-double stranded (ds)DNA and anti-Sm antibodies to assess whether this would improve the model. Asterisks indicate statistically significant associations. SRI-4: systemic lupus erythematosus responder index 4; SLEDAI-2K: Systemic Lupus Erythematosus Disease Activity Index 2000; SDI: Systemic Lupus International Collaborating Clinics (SLICC)/American College of Rheumatology (ACR) Damage Index; HR: hazard ratio; CI: confidence interval.

For analysis of the systemic lupus erythematosus responder index 4 (SRI-4) response, at least two follow-up visits, data availability allowing determination of the SRI-4 components, and a baseline SLEDAI-2K score of 4 or higher were required. Of a total of 57 patients qualifying for analysis, 39 patients (68.4%) achieved sustained SRI-4 during follow-up, after a median time of 3.4 (IQR: 2.8–7.9) months; 18 patients (31.6%) did not achieve the outcome after having been followed for a median time of 13.4 (IQR: 10.4–39.6) months. In Cox regression analysis, higher baseline SLEDAI-2K scores and prednisone equivalent doses were associated with higher probability and/or shorter time to sustained SRI-4 response (hazard ratio, HR: 1.10; 95% confidence interval, CI: 1.05–1.16; *p* < 0.001 and HR: 1.04; 95% CI: 1.01–1.07; *p* = 0.002, respectively), and higher baseline Systemic Lupus International Collaborating Clinics (SLICC)/American College of Rheumatology (ACR) Damage Index (SDI) scores were associated with lower probability and/or longer time to sustained SRI-4 response (HR: 0.76; 95% CI: 0.58–0.99; *p* = 0.044). Age, sex, SLE disease duration, and hypocomplementaemia showed no significant association with achievement of SRI-4 response (Figure 5). Notably, anti-Sm antibody positivity was associated with higher probability and/or shorter time to achieve sustained SRI-4 response (HR: 2.52; 95% CI: 1.20–5.29; *p* = 0.015); this association remained significant after adjustment for baseline SLEDAI-2K scores, prednisone equivalent doses, and baseline SDI scores, including the significant interaction between SLEDAI-2K scores and prednisone doses (Figure 5). Substituting anti-Sm positivity with positivity for both anti-dsDNA and anti-Sm (*n* = 12) slightly improved the model, yielding a HR of 3.06 (95% CI: 1.30–7.21; *p* = 0.011; Figure 5). No other cytokines or autoantibody specificities were found to impact on attainment of sustained SRI-4 response in our cohort. No association was found between SRI-4 response attainment and positive levels of circulating IC at baseline.

For analysis with regard to clinical remission, at least two follow-up visits and SLEDAI-2K and prednisone dose data availability were required. Of a total of 66 patients qualifying for analysis, 27 patients (40.9%) achieved sustained clinical version of SLEDAI-2K (cSLEDAI-2K) = 0 during follow-up, after a median time of 6.6 (IQR: 5.8–12.2) months; 39 patients (59.1%) did not achieve the outcome after having been followed for a median time of 13.1 (IQR: 9.3–36.1) months. No demographic (age, sex) or disease-associated (disease activity, organ damage, disease duration, glucocorticoid dose, C3/C4 status) characteristics and no cytokine, autoantibody specificity, or IC positivity showed any association with attainment of cSLEDAI-2K = 0 in Cox regression analysis. This was also the case when the glucocorticoid restriction (prednisone equivalent dose ≤ 7.5 mg/day) was added in the definition criteria (Figure S1). Sustained cSLEDAI-2K = 0 and prednisone equivalent dose ≤ 7.5 mg/day was achieved by 22 patients (33.3%) after a median time of 7.8 (IQR: 6.1–17.5) months, whereas 44 patients (66.7%) did not meet the criteria of this composite outcome during follow-up, that is, a median time of 13.4 (IQR: 9.7–35.7) months. 

Similarly, no significant associations were found with regard to attainment of sustained Lupus Low Disease Activity State (LLDAS; Figure S1). A total of 35/66 patients (53.0%) achieved sustained LLDAS after a median time of 7.9 (IQR: 5.0–16.4) months; 31 patients (47.0%) did not attain sustained LLDAS after a total median follow-up of 11.5 (IQR: 9.3–23.9) months.

Numbers and proportions of patients with undetectable cytokine levels or negative autoantibody or IC levels at baseline who achieved sustained SRI-4, cSLEDAI-2K = 0, cSLEDAI-2K = 0 and prednisone equivalent dose ≤ 7.5 mg/day, or LLDAS are presented in Table S2. 

### 2.5. Early Changes in Cytokine, Autoantibody, and IC Levels as Predictors of Response

Early changes in cytokine, autoantibody, and IC levels from baseline to month 3 (Table S3) and from baseline to month 6 (Table S4) were next evaluated as predictors of response to belimumab therapy. Early decline in IL-6 levels from baseline to month 3 was found to be consistently associated with attainment of sustained SRI-4, cSLEDAI-2K = 0, and cSLEDAI-2K = 0 and prednisone equivalent dose ≤ 7.5 mg/day (Table S3). In a subanalysis of patients who had detectable levels of IL-6 at baseline or month 3, IL-6 levels declined from baseline to month 3 in patients who later attained sustained cSLEDAI-2K = 0 and prednisone equivalent dose ≤ 7.5 mg/day (median change: -5.9 pg/mL; 25th percentile: -6.8 pg/mL; 75th percentile: -4.6 pg/mL), but increased in patients who did not attain this outcome (median change: 3.2 pg/mL; 25th percentile: -0.4 pg/mL; 75th percentile: 18.1 pg/mL), yielding a significant difference (*p* = 0.029).

## 3. Discussion

In the present study, we demonstrated that IFN-α2, IL-6, IL-10, circulating IC levels, and levels of multiple autoantibodies against nuclear components decreased during anti-BAFF treatment with belimumab, albeit differently in terms of intensity and/or sustainability. To the best of our knowledge, this is the first report to assess the performance of positive status regarding multiple nuclear antigen autoantibody specificities as predictors of response to belimumab treatment. Interestingly, anti-Sm antibody positivity at baseline and early decline of IL-6 levels were associated with favorable response to belimumab treatment.

Serum levels of IL-10 showed the most rapid and prominent changes, being significant from month 3 and throughout the entire follow-up period. Serum levels of IFN-α2 and IL-6 also decreased, however in a more moderate and slower fashion. The decreases in IL-10 and IL-6 levels most likely reflect the overall abatement of inflammatory activity, following the decreases in clinical markers of disease activity, as shown herein and in previous observational studies [2,4,5,17,18,19,20,21] and clinical trials [22,23,24,25]. Although the principal functions of IL-10 were initially coupled with suppression of cytokine secretion and termination of inflammatory responses [26,27], there is evidence that IL-10 may induce and amplify autoantibody production in autoimmune conditions [28,29,30], and IL-10 levels may therefore reflect the inflammatory state in such conditions. Importantly, early decline in IL-6 levels within 3 months of belimumab treatment was associated with belimumab efficacy, that is, attainment of sustained SRI-4 and clinical remission. The consistency in these associations advocate for the role of IL-6 as a useful marker of inflammation in patients with SLE, as also implied in our recent study of rituximab therapy [31]. In patients with detectable IL-6 levels at baseline, early declines could hence signify suitability for treatment continuation. 

IFN-α signaling is known to be aberrant in SLE [32]; type I IFNs derived from plasmacytoid dendritic cells (pDCs) are important in proinflammatory cytokine production, including B cell differentiation and survival factors BAFF and APRIL [33]. The mechanistic explanation underlying the decrease of IFN-α levels is not totally clear, but could be traced to the overall decrease of autoantibody levels and circulating IC, presumably resulting in reduced Fcγ receptor stimulation on pDCs towards production of type I IFNs [32]. Thus, belimumab treatment might have indirect effects on the BAFF/APRIL pathway apart from the direct binding to soluble BAFF. In light of the recent clinical trials of anti-IFN-α (rontalizumab [34], sifalimumab [35]) and anti-IFN α/β receptor (IFNAR; antifrolumab [36,37]) agents in SLE, it is of particular importance to demonstrate how a currently available lupus therapy indirectly impacts on this pathway. Nonetheless, it is important to place emphasis on the fact that IFN-α2 only represents a fraction of the type I IFN activity; studies of larger cohorts and analyses of other cytokines of the same family are therefore necessary in order to more accurately evaluate the impact of belimumab treatment on the type I IFN pathway. It is worth noting that only one patient had detectable levels of IL-17A at baseline, which contrasts with a previous report of higher proportions (12%) in SLE patients from Sweden [38], and might depend on the different assays used to measure cytokine levels in the two studies, or patient selection. Supportive of the latter is a previous report from our group that demonstrated elevated IL-17 levels in SLE patients with nephritis [39]. In the same lupus nephritis cohort, the proportion of patients with detectable INF-α levels was also higher (66%) [40] compared with the findings in the present study (15%).

Serum levels of autoantibodies belonging to the Ro/La-system, that is, anti-Ro52/SSA, anti-Ro60/SSA, and anti-La/SSB, did not display substantial changes throughout the follow-up period. In SLE, levels of these antibodies have not been shown to have a value in monitoring disease activity [41], in line with their inertia to change in our study. Serum levels of anti-dsDNA and anti-Sm antibodies showed profound, rapid, and sustainable decreases throughout the follow-up. In a similar manner, levels of anti-U1RNP and anti-Sm-U1RNP antibodies as well as IC levels decreased rapidly and remained decreased during follow-up. Ratios of anti-dsDNA, anti-ribosomal P, and anti-PCNA to total IgG also decreased, pointing to a specific effect of belimumab treatment on these specificities rather than reflection of the general effect of the drug on total IgG levels. The observation that only two patients had positive levels of anti-PCNA at baseline is worth noting in light of our recent report showing profound enrichment of this particular specificity in IC [9], pointing to high binding affinity of anti-PCNA to its autoantigen.

An important finding was that, in conformity with previous observations following B cell depletion with the anti-CD20 antibody rituximab [42], the presence of anti-Sm but not anti-dsDNA antibodies was associated with a higher probability and/or shorter time to achieve clinical improvement following belimumab treatment, irrespective of disease activity, glucocorticoid dose, and organ damage degree. These findings contrast with observations from early clinical trials of belimumab in which proportions of responders did not differ across patient subsets positive for different autoantibody subtypes at baseline, including anti-dsDNA, anti-Sm, and anti-Ro [43], and also with later studies of belimumab in which anti-dsDNA positivity was implicated to be a predictor of SRI-4 response [6], LLDAS [44], and cSLEDAI-2K = 0 and prednisone dose ≤ 7.5 mg/day [8] in different reports. The lack of association between baseline autoantibody profiles and LLDAS or clinical remission in the present study may have been due to the stringency of these outcomes along with the relatively low number of patients; as previously shown, SRI-4 is sensitive to change, whereas attainment of LLDAS and clinical remission is less frequent and occurs at later time points [4,44,45,46]. Double positivity for anti-dsDNA and anti-Sm antibodies yielded a slightly stronger association with clinical improvement compared with anti-Sm positivity alone, but because a lower number of patients is expected to be positive for both antibodies, the clinical value of this stringency augmentation is questionable. Further investigation of the clinical usefulness of anti-Sm positivity in the selection of patients for belimumab treatment is merited.

The low number of patients and, as a result, the low frequencies of detectable cytokine levels at baseline limited us from performing certain analyses. For example, stratification into clinical manifestations would be of relevance; from a clinical point of view, the therapy is steered by organ involvement, and identification of predictors within different disease subsets is needed to facilitate individualized management approaches. For the same reason, we were unable to analyze concomitant and previous medication other than glucocorticoids. The observational design may also be considered a drawback; decisions steered by the treating physician and not the purposes of the study may, for instance, impede standardisation of the background therapy. However, the patient cases represent real-life scenarios and the follow-up represents current clinical practice, both of which may also be regarded as strengths of the study. In contrast to clinical trial settings, no exclusion criteria were applied in the present study; all patients who were initiated at belimumab treatment were asked to be included in our prospective follow-up program, increasing the heterogeneity of the study cohort, and minimizing the selection bias. 

The prospective collection of detailed clinical data along with regular serum sampling may be acknowledged as a strength. It is also worth noting that the study was conducted at tertiary referral centers of the tax-financed health system of Sweden, limiting potential bias imposed by private health insurance in other systems. Investigation of multiple nuclear antigen autoantibody specificities in the context of belimumab treatment was a novelty, and the consistent level reductions of the majority of them has to be seen in light of previous knowledge that belimumab treatment alters the B cell constitution of SLE patients towards declining numbers and proportions of naïve and autoreactive B cells over time on therapy [3,16].

## 4. Materials and Methods 

A total of 78 patients with SLE from the Karolinska (*n* = 45), Skåne (*n* = 23), and Linköping (*n* = 10) University Hospitals who were initiated at belimumab treatment between September 2011 and October 2018 were included in the present real-life observational study. All patients met the 1982 ACR [47] and/or SLICC [48] criteria for classification of SLE. Belimumab was given as intravenous infusions at a dose of 10 mg/kg at baseline, week 2, week 4, and every fourth week thereafter. Baseline characteristics of the patients are summarized in Table 1.

The study complied with the ethical principles of the Declaration of Helsinki, and all study participants signed informed consent forms prior to recruitment. The study protocol was reviewed and approved by the respective regional ethics review boards in Stockholm, Lund, and Linköping.

### 4.1. Determination of Levels of Cytokines, Autoantibodies and IC

Serum samples obtained at baseline and after 3, 6, 12, and 24 months of treatment were stored at −80 °C until analysis. All samples were obtained prior to belimumab infusion. Data from clinical and laboratory evaluations from the same time points were incorporated in the dataset.

Serum cytokine levels, that is, IFN-α2, IL-6, IL-10, and IL-17A, were measured using Luminex xMAP technology (Milliplex Map kit HCYTOMAG-60K-04; EMD Millipore Corp., Billerica, USA). The corresponding serum concentrations were expressed in pg/mL. The lower detection limit of the assay was 1.5 pg/mL for IFN-α2, 0.5 pg/mL for IL-6, 0.6 pg/mL for IL-10, and 0.4 pg/mL for IL-17A. Non-detectable levels were set to half the lower detection limit for the purpose of statistical analysis. 

Serum levels of nuclear antigen autoantibodies against dsDNA, tripartite motif-containing protein 21 (TRIM21, or Ro52/SSA), Ro60/SSA, La/SSB, Smith antigen (Sm), the Sm-U1RNP complex, U1RNP, histone, ribosomal P, and proliferating cell nuclear antigen (PCNA), were determined by addressable laser bead immunoassay (ALBIA) using the Connective Profile MX117 FIDISTM kit (Theradiag, Croissy Beaubourg, Marne La Vallée, France). Serum levels of antibodies were expressed in arbitrary units per mL (AU/mL), except for anti-dsDNA levels which were expressed in international units per mL (IU/mL). Data evaluation was performed using the Theradiag Solinium software. Levels over 40 AU/mL or IU/mL were considered positive, as recommended by the manufacturer. Serum levels of total IgG were determined using an in-house enzyme-linked immunosorbent assay (ELISA), as described in previous studies [9,50,51], and expressed as mg/mL.

Serum concentrations of C1q-binding IC were measured using Quanta Lite ELISA (INOVA Diagnostics, San Diego, CA, USA). Serum IC concentrations were expressed as microgram equivalents per milliliter (μg Eq/mL), and levels over 10.8 μg Eq/mL were considered positive. Complement C3 (reference range 0.67–1.29 g/L) and C4 (reference range 0.13–0.32 g/L) levels were determined using nephelometry at the local laboratory of each one of the centers.

### 4.2. Clinical Evaluation

Clinical assessment was performed at baseline, month 3, 6, 12, 24, and thereafter once yearly until month 72, or at clinical indication. The total follow-up time for each one of the patients is visualized in Figure 1. Reasons for observations at early time points only included adverse events and recent recruitment. Reasons for discontinuation at late time points included inadequate effect, adverse events, pregnancy plans, and disease quiescence, as previously reported for the majority of patients [4].

We assessed global SLE disease activity using the SLEDAI-2K [52], and organ damage using the SLICC/ACR Damage Index (SDI) [53]. For SLEDAI-2K scores, laboratory and serological items were calculated on the basis of routine test results at the local laboratories; for the anti-dsDNA item, the *Crithidia luciliae* substrate-based immunofluorescence technique (CLIFT) [54] was used.

We defined response to treatment as attainment of the SLE Responder Index (SRI)-4 criteria [22,23], that is, reduction of ≥4 points in the SLEDAI-2K score, no new A, and no more than one new B in the British Isles Lupus Assessment Group (BILAG) index [55], and no worsening in the physician’s global assessment (PGA) by ≥30% compared with the baseline evaluation. We defined clinical remission as a zero score in the clinical version of SLEDAI-2K (cSLEDAI-2K), in which the serological items (anti-dsDNA and complement levels) are excluded [56]. Finally, we defined low disease activity in accordance with the Lupus Low Disease Activity State (LLDAS) [57], requiring a SLEDAI-2K score ≤4 with no activity in major organ systems (renal activity, central nervous system involvement, cardiopulmonary activity, vasculitis, fever), no signs of haemolytic anaemia or gastrointestinal activity, no new features of SLE disease activity, a physician’s global assessment (PGA) score ≤1 (on a scale 0–3), a prednisone or prednisone equivalent dose of ≤ 7.5 mg/day, and well-tolerated doses of immunosuppressive drugs and/or approved biologic agents.

According to recent treat-to-target recommendations, treatment should aim for sustained clinical remission or low disease activity with the lowest glucocorticoid dose possible [58]. In order to incorporate the latter in the definition of clinical remission used here, we next calculated a composite clinical remission score in which a glucocorticoid dose restriction was added to cSLEDAI-2K = 0, that is, a prednisone or prednisone equivalent dose of ≤ 7.5 mg/day, as used in previous studies [8,45]. For each one of the aforementioned definitions, we further required that the patient met the respective definition criteria at the evaluation of at least two consecutive follow-up visits, at least 3 months apart, and therefore termed the definitions “sustained SRI-4”, “sustained cSLEDAI-2K = 0”, “sustained cSLEDAI-2K = 0 and prednisone equivalent dose ≤ 7.5 mg/day”, and “sustained LLDAS”. For the purpose of proportional hazards (Cox) regression analysis, time from belimumab treatment initiation until achievement of each one of the sustained definitions was registered.

### 4.3. Statistics

The IBM SPSS Statistics 25 software (IBM Corp., Armonk, NY, USA) was used for statistical analyses. Comparisons between baseline and follow-up visits were conducted using the non-parametric paired Wilcoxon signed rank test. Proportional hazards (Cox) regression models were created in order to evaluate the potential associations of baseline cytokine and autoantibody or IC levels (detectable and positive levels, respectively) with time-varying achievement of predefined outcomes (sustained SRI-4, clinical remission, and LLDAS). Demographic (age, sex) and disease-specific (disease activity, organ damage, disease duration, C3/C4 status) characteristics were included in multivariable models together with cytokines and autoantibodies under investigation, in case they reached statistical significance in initial univariable models. Associations between early changes in cytokine, autoantibody, or IC levels and predefined outcomes were evaluated using the non-parametric Mann–Whitney *U* test. *p* values < 0.05 were considered statistically significant. Graphs were constructed using the GraphPad Prism 7 software.

## 5. Conclusions

In our cohort, belimumab treatment lowered IFN-α2, IL-6, IL-10, and circulating IC levels, as well as levels of multiple autoantibodies against nuclear components, however, after different time spans from baseline. Notably, anti-Sm antibody positivity at baseline and early decline of IL-6 levels were associated with favorable response to belimumab treatment independently of other factors. Further investigation of these associations is merited.

## Figures and Tables

**Table 1:** Patient characteristics.

Baseline Characteristics	Value
Female sex; *n* (%)	73 (93.6)
Age (years); M (IQR)	41.3 (31.6–51.4)
Ancestry	
European/Caucasian; *n* (%)	71 (91.0)
African/African American; *n* (%)	5 (6.4)
Hispanic; *n* (%)	2 (2.6)
SLE disease duration (years); M (IQR)	7.5 (3.7–13.3)
SLEDAI-2K score; M (IQR)	8.0 (4.0–12.0)
Number of DMARDs *; M (IQR)	1 (0–1); *N* = 77
Azathioprine; *n* (%)	21 (27.3); *N* = 77
Mycophenolate; *n* (%)	11 (14.3); *N* = 77
Methotrexate; *n* (%)	14 (18.2); *N* = 77
Cyclosporine; *n* (%)	3 (3.9); *N* = 77
Antimalarial agents; *n* (%)	57 (74.0); *N* = 77
Corticosteroid use; *n* (%)	71 (91.0)
Previous exposure to corticosteroids (years); M (IQR)	6.6 (3.6–11.0); *N* = 54
Previous mean prednisone equivalent (mg/day); M (IQR)	10.0 (7.5–12.5); *N* = 55
Reason for belimumab	
Arthritis; *n* (%)	39 (52.7); *N* = 74
Mucocutaneous manifestations; *n* (%)	39 (52.7); *N* = 74
Hematological manifestations; *n* (%)	10 (13.5); *N* = 74
Lupus nephritis; *n* (%)	7 (9.5); *N* = 74
Neuropsychiatric lupus; *n* (%)	4 (5.4); *N* = 74
Serositis; *n* (%)	3 (4.1); *N* = 74
General manifestations ^†^; *n* (%)	3 (4.1); *N* = 74
Serologic activity; *n* (%)	1 (1.4); *N* = 74
Respiratory ^‡^; *n* (%)	1 (1.4); *N* = 74

Data are presented as numbers (*n*) and percentages (%), or medians (M) and interquartile ranges (IQR). The total number of patients was 78; in cases of missing data, the total number of observations (*N*) is indicated. * Excluding antimalarial agents. † Fatigue. ‡ Lung bleeding prophylaxis [49]. SLE: systemic lupus erythematosus; SLEDAI-2K: SLE Disease Activity Index 2000; DMARDs: disease-modifying antirheumatic drugs.

## Supplementary Materials

Supplementary materials can be found at https://www.mdpi.com/1422-0067/21/10/3463/s1.
