# Supplementary material for: Autoantibody and Cytokine Profiles during Treatment with Belimumab in Patients with Systemic Lupus Erythematosus
Source: Int J Mol Sci. 2020 May 14;21(10):3463. doi: 10.3390/ijms21103463 (PMC7278961; doi:10.3390/ijms21103463)
Supplement: Supplementary file 1 [file ijms-21-03463-s001.zip › Supplementary Table S1.pdf]

Table S1. Seroconversion of patients with undetectable cytokine or negative autoantibody or immune complex levels at baseline.

	Undetectable or negative at baseline	Month 3	Month 6	Month 12	Month 24
**Cytokines**					
IFN-α2	63	0	1	0	0
IL-6	62	1	2	2	0
IL-10	50	1	1	3	0
IL-17A	72	3	1	0	1
**Autoantibodies**					
Anti-dsDNA	32	0	1	3	0
Anti-ribosomal P	63	1	0	1	1
Anti-histone	59	0	0	3	2
Anti-Sm	58	0	0	1	0
Anti-U1RNP	43	0	3	2	0
Anti-Sm-U1RNP	59	0	0	0	0
Anti-Ro52/SSA	46	0	1	2	0
Anti-Ro60/SSA	33	0	1	3	0
Anti-La/SSB	59	0	0	1	0
Anti-PCNA	72	0	0	0	0
**Circulating IC**	57	1	1	3	2

Numbers of patients with undetectable cytokine levels or negative autoantibody or immune complex levels at baseline who seroconverted to detectable cytokine levels or positive autoantibody levels, respectively, at later time points.

dsDNA: double stranded DNA; IFN: interferon; IC: immune complex; IL: interleukin; PCNA: proliferating cell nuclear antigen; Sm: Smith.
