# Supplementary material for: Autoantibody and Cytokine Profiles during Treatment with Belimumab in Patients with Systemic Lupus Erythematosus
Source: Int J Mol Sci. 2020 May 14;21(10):3463. doi: 10.3390/ijms21103463 (PMC7278961; doi:10.3390/ijms21103463)
Supplement: Supplementary file 1 [file ijms-21-03463-s001.zip › Supplementary Table S2.pdf]

Table S2. Proportions of responders among patients with undetectable cytokine or negative autoantibody or immune complex levels at baseline.

Sustained outcomes	SRI-4	cSLEDAI-2K=0	cSLEDAI-2K=0 & prednisone eq. ≤7.5 mg/day	LLDAS
**Cytokines**				
IFN-α2	31/46 (67.4%)	24/54 (44.4%)	19/54 (35.2%)	29/54 (53.7%)
IL-6	27/44 (61.4%)	21/52 (40.4%)	17/52 (32.7%)	25/52 (48.1%)
IL-10	24/36 (66.7%)	18/43 (41.9%)	13/43 (30.2%)	23/43 (53.5%)
IL-17A	36/55 (65.5%)	26/63 (41.3%)	21/63 (33.3%)	32/63 (50.8%)
**Autoantibodies**				
Anti-dsDNA	13/24 (54.2%)	13/28 (46.4%)	10/28 (35.7%)	15/28 (53.6%)
Anti-ribosomal P	33/46 (71.7%)	25/53 (47.2%)	20/53 (37.7%)	30/53 (56.6%)
Anti-histone	27/42 (64.3%)	21/50 (42.0%)	17/50 (34.0%)	28/50 (56.0%)
Anti-Sm	28/46 (60.9%)	23/49 (46.9%)	20/49 (40.8%)	26/49 (53.1%)
Anti-U1RNP	20/35 (57.1%)	19/39 (48.7%)	16/39 (41.0%)	17/39 (43.6%)
Anti-Sm-U1RNP	28/46 (60.9%)	23/51 (45.1%)	19/51 (37.3%)	26/51 (51.0%)
Anti-Ro52/SSA	24/37 (64.9%)	19/41 (46.3%)	16/41 (39.0%)	22/41 (53.7%)
Anti-Ro60/SSA	17/26 (65.4%)	13/29 (44.8%)	11/29 (37.9%)	15/29 (51.7%)
Anti-La/SSB	29/45 (64.4%)	21/51 (41.2%)	17/51 (33.3%)	25/51 (49.0%)
**Circulating IC**	25/41 (61.0%)	21/49 (42.9%)	16/49 (32.7%)	28/49 (57.1%)

Numbers (percentage) of patients with undetectable cytokine levels or negative autoantibody or immune complex levels at baseline who achieved sustained SRI-4, cSLEDAI-2K = 0, cSLEDAI-2K = 0 & prednisone equivalent dose ≤ 7.5 mg/day, and LLDAS.

cSLEDAI-2K: clinical Systemic Lupus Erythematosus Disease Activity Index 2000; dsDNA: double stranded DNA; IFN: interferon; IC: immune complex; IL: interleukin; LLDAS: Lupus Low Disease Activity State; PCNA: proliferating cell nuclear antigen; Sm: Smith.
