# Supplementary material for: Autoantibody and Cytokine Profiles during Treatment with Belimumab in Patients with Systemic Lupus Erythematosus
Source: Int J Mol Sci. 2020 May 14;21(10):3463. doi: 10.3390/ijms21103463 (PMC7278961; doi:10.3390/ijms21103463)
Supplement: Supplementary file 1 [file ijms-21-03463-s001.zip › Supplementary Table S3.pdf]

Table S3. Associations of changes in cytokine, autoantibody and IC levels between baseline and month 3 with treatment outcome.

Sustained outcomes	SRI-4			cSLEDAI-2K=0			cSLEDAI-2K=0 & prednisone eq. ≤7.5 mg/day			LLDAS		
**Variables**	+	-	P value	+	-	P value	+	-	P value	+	-	P value
**Cytokines**												
IFN-α2			0.176			0.113			0.179			0.074
IL-6*	0.0; -0.8 (0.0; 0.0)	0.0; 1.8 (0.0; 0.0)	0.043	0.0; -1.0 (0.0; 0.0)	0.0; 1.1 (0.0; 0.0)	0.004	0.0; -1.1 (0.0; 0.0)	0.0; 0.9 (0.0; 0.0)	0.017			0.170
IL-10			0.547			0.834			0.743			0.691
IL-17A			0.295			0.660			0.868			0.096
**Autoantibodies**												
Anti-dsDNA			0.165			0.843			0.855			0.692
Anti-ribosomal P			0.959	-1.3 (-11.1; 0.0)	-1.0 (-18.0; -0.2)	0.037			0.103			0.099
Anti-histone			0.929			0.925			0.524			0.591
Anti-Sm			0.759			0.484			0.085			0.312
Anti-U1RNP	-4.7 (-14.2; -0.3)	-0.2 (-3.1; 0.6)	0.033			0.157			0.289			0.406
Anti-Sm-U1RNP			0.267	-0.1 (-5.5; 0.0)	-1.5 (-9.0; -0.2)	0.044			0.051			0.977
Anti-Ro52/SSA			0.157			0.873			0.885			0.902
Anti-Ro60/SSA			0.610			0.713			0.196			0.138
Anti-La/SSB			0.251			0.439			0.596			0.160
**Circulating IC**			0.074			0.775			0.398			0.516

P values derived from comparisons of changes in cytokine (pg/mL), autoantibody (AU or IU/mL) and IC (μg Eq/mL) levels between baseline and month 3 ($\Delta_{(\text{month 3 - baseline})}$) between patients who attained and patients who did not attain sustained SRI-4, cSLEDAI-2K = 0, cSLEDAI-2K = 0 & prednisone equivalent dose ≤ 7.5 mg/day, and LLDAS, using Mann-Whitney *U* test. Distributions of variables are presented as medians (25th percentile; 75th percentile) in cases of significant P values. Light grey shadows indicate statistically significant associations between Δs and the respective outcome.

* For IL-6, means are also provided to determine the direction and magnitude of the change due to zero median values.

cSLEDAI-2K: clinical Systemic Lupus Erythematosus Disease Activity Index 2000; dsDNA: double stranded DNA; IFN: interferon; IC: immune complex; IL: interleukin; LLDAS: Lupus Low Disease Activity State; PCNA: proliferating cell nuclear antigen; Sm: Smith.
