# Supplementary material for: Autoantibody and Cytokine Profiles during Treatment with Belimumab in Patients with Systemic Lupus Erythematosus
Source: Int J Mol Sci. 2020 May 14;21(10):3463. doi: 10.3390/ijms21103463 (PMC7278961; doi:10.3390/ijms21103463)
Supplement: Supplementary file 1 [file ijms-21-03463-s001.zip › Supplementary Table S4.pdf]

Table S4. Associations of changes in cytokine, autoantibody and IC levels between baseline and month 6 with treatment outcome.

Sustained outcomes	SRI-4	cSLEDAI-2K=0	cSLEDAI-2K=0 & prednisone eq. ≤7.5 mg/day	LLDAS
**Variables**	+ -<br>**P value**	+ -<br>**P value**	+ -<br>**P value**	+ -<br>**P value**
**Cytokines**				
IFN-α2	1.000	0.397	0.356	0.716
IL-6	0.216	0.172	0.147	0.149
IL-10	0.957	0.358	0.269	0.541
IL-17A	0.894	0.622	0.638	0.517
**Autoantibodies**				
Anti-dsDNA	0.362	0.612	0.876	0.198
Anti-ribosomal P	0.669	0.717	0.950	0.064
Anti-histone	0.206	0.852	0.876	-0.8 (-2.6; -0.1) -6.2 (-19.6; -0.6)<br>0.031
Anti-Sm	0.391	0.192	0.160	-0.1 (-3.5; 0.9) -1.0 (-25.8; -0.2)<br>0.013
Anti-U1RNP	0.453	-0.5 (-6.6; -0.1) -5.1 (-31.4; -0.2)<br>0.040	0.072	0.341
Anti-Sm-U1RNP	0.061	0.069	0.124	-0.2 (-5.3; 0.1) -3.3 (-15.4; -0.4)<br>0.038
Anti-Ro52/SSA	0.138	0.649	0.678	0.594
Anti-Ro60/SSA	0.105	0.066	-0.5 (-2.8; 1.9) -2.7 (-7.0; -0.2)<br>0.031	-0.2 (-3.3; 2.1) -2.8 (-7.1; -1.0)<br>0.011
Anti-La/SSB	0.238	0.357	0.454	0.0 (-1.2; 0.3) -0.9 (-3.7; -0.1)<br>0.028
**Circulating IC**	0.138	0.381	0.599	0.437

P values derived from comparisons of changes in cytokine (pg/mL), autoantibody (AU or IU/mL) and IC (μg Eq/mL) levels between baseline and month 6 ($\Delta_{(month\ 6\ -\ baseline)}$) between patients who attained and patients who did not attain sustained SRI-4, cSLEDAI-2K = 0, cSLEDAI-2K = 0 & prednisone equivalent dose ≤ 7.5 mg/day, and LLDAS, using Mann-Whitney *U* test. Distributions of variables are presented as medians (25th percentile; 75th percentile) in cases of significant P values. Light grey shadows indicate statistically significant associations between Δs and the respective outcome.

cSLEDAI-2K: clinical Systemic Lupus Erythematosus Disease Activity Index 2000; dsDNA: double stranded DNA; IFN: interferon; IC: immune complex; IL: interleukin; LLDAS: Lupus Low Disease Activity State; PCNA: proliferating cell nuclear antigen; Sm: Smith.
