# Supplementary material for: Autoantibody and Cytokine Profiles during Treatment with Belimumab in Patients with Systemic Lupus Erythematosus
Source: Int J Mol Sci. 2020 May 14;21(10):3463. doi: 10.3390/ijms21103463 (PMC7278961; doi:10.3390/ijms21103463)
Supplement: Supplementary file 1 [file ijms-21-03463-s001.zip › Figure S1 caption.pdf]

Figure S1. Associations with clinical remission and low disease activity. The forest plots illustrate results from proportional hazards regression (Cox regression) models created to explore potential associations between baseline levels of cytokines, autoantibodies, or circulating immune complexes (IC), and achievement of sustained clinical (c)SLEDAI-2K = 0, sustained cSLEDAI-2K = 0 and prednisone equivalent dose ≤ 7.5 mg/day, or sustained LLDAS. Demographic and disease-specific factors were also analyzed for the purpose of adjustments for confounding potentiality. Time in the models represents the time from baseline to the first follow-up visit when the outcome was achieved for patients who achieved the outcome, and total follow-up time for patients who did not. No significant associations were found in univariable (simple) models; therefore, no multivariable analysis was performed. SRI-4: Systemic lupus erythematosus Responder Index 4; SLEDAI-2K: Systemic Lupus Erythematosus Disease Activity Index 2000; LLDAS: Lupus Low Disease Activity State; SDI: Systemic Lupus International Collaborating Clinics (SLICC)/American College of Rheumatology (ACR) Damage Index; HR: hazard ratio; CI: confidence interval.
